# Plasma-derived exosomal let-7c-5p, miR-335–3p, and miR-652–3p as potential diagnostic biomarkers for stable coronary artery disease

**DOI:** 10.3389/fphys.2023.1161612

**Published:** 2023-05-09

**Authors:** Jian Han, Xiaogang Cui, Tianqi Yuan, Zhiming Yang, Yue Liu, Yajuan Ren, Changxin Wu, Yunfei Bian

**Affiliations:** ^1^ Third Hospital of Shanxi Medical University, Shanxi Bethune Hospital, Shanxi Academy of Medical Sciences, Tongji Shanxi Hospital, Taiyuan, China; ^2^ Department of Cardiology, The Second Hospital of Shanxi Medical University, Taiyuan, China; ^3^ Key Lab of Medical Molecular Cell Biology of Shanxi Province, Institutes of Biomedical Sciences, Shanxi University, Taiyuan, China; ^4^ Key Laboratory of Chemical Biology and Molecular Engineering of Ministry of Education, Shanxi University, Taiyuan, China

**Keywords:** stable coronary artery disease, plasma, exosomes, microRNA, biomarker

## Abstract

**Objectives:** Circulating exosomal microRNAs (miRNAs) have been identified as promising biomarkers for diagnosis of cardiovascular diseases. Nevertheless, the diagnostic potential of miRNAs in circulating exosomes for stable coronary artery disease (SCAD) remains unclear. We aim here to analyze the exosomal differentially expressed miRNAs (DEmiRNAs) in plasma of SCAD patients and investigate their diagnostic potential as SCAD biomarkers.

**Methods:** Plasma was collected from SCAD patients and healthy controls, and exosomes were isolated by ultracentrifugation. Exosomal DEmiRNAs were analyzed by small RNA sequencing and were further validated by quantitative real-time PCR (qRT-PCR) in a larger set of plasma samples. Relationships between plasma exosomal let-7c-5p, miR-335–3p, miR-652–3p, genders and Gensini Scores in patients with SCAD were analyzed using correlation analyses. Moreover, we conducted receiver operating characteristic (ROC) curves for these DEmiRNAs and analyzed their possible functions and signaling pathways.

**Results:** Vesicles isolated from plasma displayed all characteristics of exosomes. In the small RNA sequencing study, a total of 12 DEmiRNAs were identified, among which seven were verified to be statistically significant by qRT-PCR. The areas under the ROC curves of exosomal let-7c-5p, miR-335–3p, and miR-652–3p were 0.8472, 0.8029, and 0.8009, respectively. Exosomal miR-335–3p levels were positively correlated with Gensini scores of patients with SCAD. Bioinformatics analysis revealed that these DEmiRNAs may be involved in the pathogenesis of SCAD.

**Conclusion:** Our findings indicated that plasma exosomal let-7c-5p, miR-335–3p, and miR-652–3p can be used as promising biomarkers for diagnosis of SCAD. In addition, plasma exosomal miR-335–3p levels coordinated with severity of SCAD.

## 1 Introduction

Coronary artery disease (CAD), one category of cardiac diseases, remains the leading cause of mortality worldwide because of its delayed diagnosis and severe complications (Virani et al., 2021). CAD can be classified into acute coronary syndrome (ACS) and stable CAD (SCAD) according to different clinical manifestations and treatment approaches. ACS is characterized by angina lasting over half-hour, coupled with an abnormal electrocardiogram (ECG) and a significant increase in troponin. As a result, ACS can be diagnosed easily based on clinical symptoms, ECG, and laboratory results. Compared to ACS, SCAD is difficult to be diagnosed due to transient angina and abnormal ECG are difficult to record at the right time. More importantly, if SCAD cannot be timely and effectively controlled, it will develop to ACS, leading to the increased risk of more severe clinical syndrome and death of patients (Virani et al., 2021).

Up to now, coronary angiography (CAG) remains the gold standard for clinical SCAD diagnosis, but it is invasive and expensive, which limit its application. Although some proteins, such as high-sensitivity cardiac troponin, creatine kinase-MB, leukocyte counts, and C-reactive proteins, are currently being used as biomarkers for diagnosis of SCAD (Lobbes et al., 2010), the diagnostic worth of these proteins remains restricted (Zhang et al., 2018). Therefore, reliable and innovative biomarkers with high precision for the early diagnosis of SCAD are urgently needed.

Exosomes, a type of extracellular vesicles, are characterized by a tea tray-like bilayer membrane structure. Exosomes contain different bioactive molecules including proteins, lipids, coding, and noncoding RNAs (ncRNAs) and play crucial roles in cell-cell communication (Isaac et al., 2021). These vesicles can be obtained from almost all body fluids and are secreted by nearly all cell types (Kalluri and LeBleu, 2020), making them rational noninvasive biomarkers for diagnosis of various diseases (Wang et al., 2021).

MicroRNAs (miRNAs) are single-stranded, small and highly evolutionarily conserved ncRNAs that are essential post-transcriptional modulators of gene expression (Ali Syeda et al., 2020). Researches have demonstrated that miRNAs in exosomes are enriched and non-degradable (Ingenito et al., 2019). In addition, there are growing evidences showing that exosomal miRNAs in the circulation may serve as promising noninvasive biomarkers for diagnosis of CAD. For example, miR-30e and miR-92a in plasma exosomes are considered as promising biomarkers for diagnosis of coronary atherosclerosis (Wang et al., 2019). MiR-183 can be used as diagnostic biomarker for myocardial ischemic injury (Zhao et al., 2019), and miR-208a, miR-133a, miR-499–5p, and miR-30a may serve as potential biomarkers for early diagnosis of acute myocardial infarction (Moreira-Costa et al., 2021). Furthermore, miR-21 and miR-126 in serum exosomes may serve as possible biomarkers for ACS diagnosis (Ling et al., 2020).

Nevertheless, few studies have focused on the possibility of plasma exosomal miRNAs as diagnostic biomarkers for SCAD. To date, only one study reported that miR-32–5p, miR-942–5p and miR-149–5p in serum exosomes could be used as promising biomarkers for diagnosis of SCAD (Zhang et al., 2020), but merely 8 miRNAs that were reported to participate in the pathogenesis of CAD were chosen as candidate miRNAs to be investigated. Hence, further research is needed to analyze the differentially expressed miRNAs (DEmiRNAs) profile and assess their diagnostic potential as SCAD biomarkers. In this study, our aim was to investigate the DEmiRNAs of plasma exosomes between SCAD patients and healthy controls and then analyze their potential diagnostic value for SCAD. Moreover, we predicted the target genes of these DEmiRNAs and analyzed their possible functions and related pathways by using bioinformatics software.

## 2 Materials and methods

### 2.1 Participants

In this study, a total of 39 SCAD patients and 39 healthy controls were recruited. All blood samples were collected from January to June 2022 from inpatients in the Department of Cardiology and healthy controls who carried out physical examinations at the Second Hospital of Shanxi Medical University in Taiyuan, China. Age, sex, and the baseline laboratory data were recorded for all the subjects. The inclusion criteria for SCAD group are as follows (Members. et al., 2013): 1) luminal stenosis ≥50% in the left main stem, or ≥70% in one or more major coronary arteries. 2) chest distress/pain lasts less than 10 min. Age- and sex-matched healthy adults who have normal ECG and no clinical manifestations of CAD were chosen to be the healthy controls. The exclusion criteria for subjects included: infection, presence of inflammation, severe hepatic or renal dysfunction, autoimmune diseases, and malignancy diseases history.

### 2.2 Sample collection and plasma isolation

Peripheral blood samples (5 mL) were collected in blood collection tube containing EDTA from the SCAD patients at 7 a.m. the day after admission and from the healthy controls before breakfast. The plasma fractions were separated from the blood samples immediately by centrifugation at 4°C, 3,000 g for 15 min and stored in RNAse-free EP tube at −80°C.

### 2.3 Exosome extraction

The plasma samples were thawed and centrifuged at 300 *g* for 10 min, 2000 g for 15 min, then at 10,000 g for 30 min. The supernatant was further ultracentrifuged at 110,000 g for 5 h, the precipitates were washed by resuspending in 9 mL of 1 × PBS and ultracentrifuged under the same condition for another 5 h. After discarding the supernatant, the precipitate was then dissolved in 200 μL 1× PBS and stored in RNAse-free EP tubes at −80°C. The centrifugation was conducted at 4°C.

### 2.4 Transmission electron microscope

The exosome suspension (5 µL) was diluted to 10 µL using 1 × PBS and absorbed onto a copper grid coated with formvar for more than 15 min. Then, the exosomes on the copper grid were negatively stained with 10 µL of a 2% phosphotungstate solution for 1 min, the residual liquid was subsequently blotted by filter paper. Several minutes later, the copper grids were then observed under an transmission electron microscope at 100 kV.

### 2.5 Nanoparticle tracking analysis

The exosome suspension (5 µL) was diluted to 30 µL using 1 × PBS and analyzed for size distribution using NanoFCM (Flow NanoAnalyzer, Xiamen, China) according to the manufacturer’s instructions.

### 2.6 Western blotting

The exosome suspension was lysed on ice with RIPA lysis buffer (Solarbio, Beijing, China) containing 1% phenylmethanesulfonyl fluoride (PMSF; Solarbio, Beijing, China), and the protein concentrations were measured using a bicinchoninic acid protein assay kit (BCA; Solarbio, Beijing, China). Protein samples (15 µg) were electrophoresed by SDS-PAGE and transferred onto nitrocellulose membranes at 300 mA. After being blocked with 5% milk in Tris-buffered saline containing 0.1% Tween-20 for 2 h at 4°C, the membrane was incubated with anti-CD9 (Abcam, ab236630, 1:1,000 dilution), anti-CD63 (Abcam, ab134045, 1:1,000 dilution), anti-Hsp70 (Abcam, ab181606, 1:1,000 dilution), and anti-Calnexin (Abcam, ab133615, 1:1,000 dilution) overnight at 4°C. Then, the membranes were incubated with the specific horseradish peroxidase-conjugated goat anti-rabbit IgG secondary antibody (Bioss, Beijing, China, 1:5,000 dilution) for 1 h at 37°C. Finally, the protein bands were visualized by chemiluminescence using a SuperKing^™^ Hypersensitive luminescent ELC solution (Abbkine, Beijing, China) and quantified by the ImageJ software.

### 2.7 Construction of small RNA library and RNA sequencing

Total RNA from the plasma exosomes was extracted using an exoRNeasy Serum/Plasma Midi Kit (Qiagen, Germany) according to the manufacturer’s instructions. The construction of small RNA library and RNA sequencing (RNA-Seq) were performed by Beijing Genomic Institute (BGI, Beijing, China). Briefly, small RNAs of 18–30 nt were purified with PAGE gel and then combined with 3′and 5′Adapter respectively. The cDNAs of the purified small RNAs were synthesized by reverse transcription reaction and were further amplified by PCR reaction. The PCR products were purified by PAGE gel and denatured to single-stranded DNAs. Single-stranded cyclized DNA molecules were produced by using a circularization reaction program and replicated to be DNA nanoballs (DNBs) *via* rolling cycle amplification. Sufficient quality DNBs were then loaded into patterned nanoarrays using high-intensity DNA nanochip technique and sequenced through combinatorial Probe-Anchor Synthesis.

The low quality tags, tags without 3′primer and insertion, tags with 5′primer contaminants and poly A, tags shorter than 15 nt were removed from the raw sequencing data to get clean tags. The clean tags were mapped to the reference genome and other sRNA databases such as siRNA, miRbase, piRNA and snoRNA with Bowtie2 (Langmead et al., 2009). The DEmiRNAs were analyzed using the DESeq2 (Love et al., 2014) on Dr. Tom Multi-omics Data Mining System (https://biosys.bgi.com) developed by BGI. A value of *q* < 0.05 was used to judge the significance of expression difference.

### 2.8 Polyadenylation and reverse transcription of miRNA

The purity of the total RNA were determined from the OD 260/280 readings between 1.8 and 2.0 using a Nanodrop 2,000 spectrophotometer (Thermo, Waltham, MA, United States). The cDNAs were synthesized using a miRcute Plus miRNA First-Strand cDNA Kit (Tiangen, Beijing, China) according to the manufacturer’s protocol. The total reaction volume was 20 μL, including 10 µL of 2 × miRNA RT Reaction Buffer, 2 µL of miRNA RT Enzyme Mix, and 8 µL of total RNA. The reaction was performed as follows: 42°C for 60 min, 95°C for 3 min.

### 2.9 Verification of the exosomal DEmiRNAs with quantitative real-time PCR

The expression levels of the exosomal DEmiRNAs obtained from the small RNA-Seq were determined by using a miRcute Plus miRNA qPCR Kit (SYBR Green) (Tiangen, Beijing, China) on a LightCycler 480 II Real-Time PCR System (LightCycler, Indianapolis, IN, United States). The parameters of the qPCR program were as follows: 95°C for 15 min, 45 cycles of 94°C for 20 s, 60°C for 34 s. The samples were run in triplicate. The miRNA sequence used for quantitative real-time PCR (qRT-PCR) are listed in Supplementary Table S1. The relative expression levels of the DEmiRNAs were normalized to U6 and expressed as 2^−ΔΔCT^.

### 2.10 Bioinformatics analysis

The target genes of the seven DEmiRNAs verified by qRT-PCR were predicted using TargetScan, RNAhybrid and miRanda. The molecular functions and signal pathways of the predicted target genes were analyzed by using online software: GO (http://www.geneontology.org/) and KEGG (https://www.genome.jp/kegg/).

### 2.11 Statistical analysis

The normal variables were analyzed using two-tailed Student’s t-test and presented as mean ± standard deviation, and nonnormal variables were analyzed using nonparametric test and presented as median with interquartile range. Relationships between continuous variables were analyzed by using Spearman’s correlation analysis. In addition, the DEmiRNAs were subjected to receiver operating characteristic (ROC) curves, and the diagnostic value of the DEmiRNAs for SCAD was evaluated by calculating the area under the ROC curve (AUC). SPSS 26.0 was used for statistical analysis. *P* < 0.05 was considered significant.

## 3 Results

### 3.1 Clinical characteristics of all the subjects

The clinical data of all the subjects involved in our study are shown in Table 1. Compared to the healthy control group, systolic blood pressure, total cholesterol, and glucose were significantly higher among the SCAD group (*p* < 0.05). No significant statistic differences in other clinical characteristics between the SCAD group and control group were observed (*p* > 0.05).

**TABLE 1:** Clinical characteristics of all the study subjects.

Variable	Small RNA sequencing phase		Verification phase	
	Control (n = 3)	SCAD (n = 3)	Control (n = 36)	SCAD (n = 36)
Sex (M/F)	2/1	2/1	22/14	24/12
Age (years)	59.67 ± 2.52	59.33 ± 1.53	58.33 ± 4.32	57.58 ± 5.74
BMI (kg/m2)	23.79 ± 1.66	24.51 ± 1.87	24.63 ± 1.62	25.02 ± 2.62
Hypertension, n (%)	1 (33.33)	1 (33.33)	7 (19.44)	12 (33.33)
Diabetes, n (%)	0 0)	0 0)	5 (13.89)	11 (30.56)
Smokers, n (%)	1 (33.33)	1 (33.33)	10 (27.78)	14 (38.89)
SBP (mmHg)	123.00 ± 19.97	134.67 ± 20.23	126.17 ± 9.90	134.08 ± 16.65*
DBP (mmHg)	78.33 ± 4.16	80.33 ± 5.51	82.53 ± 5.49	83.75 ± 11.28
TC (mmol/L)	3.78 ± 0.93	4.06 ± 0.59	3.70 ± 0.99	4.20 ± 0.91*
TG (mmol/L)	1.55 ± 0.82	1.72 ± 0.26	1.63 ± 0.68	2.01 ± 1.37
LDL-C (mmol/L)	2.66 ± 0.51	2.27 ± 0.71	2.61 ± 0.63	2.32 ± 0.74
Glucose (mmol/L)	4.57 ± 0.39	4.95 ± 0.20	5.03 ± 0.70	5.72 ± 1.52*
hs-cTnI (pg/mL)	1.87 ± 0.25	2.00 ± 0.98	3.37 ± 1.46	4.71 ± 3.96
ALT (U/L)	15.67 ± 4.38	18.47 ± 4.24	20.50 ± 5.48	23.11 ± 7.17
AST (U/L)	18.13 ± 3.89	21.17 ± 1.53	20.08 ± 4.92	21.15 ± 5.14

Data are presented as mean ± SD. SCAD: stable coronary artery disease; M/F: male/female; BMI: body mass index; SBP: systolic blood pressure; DBP: diastolic blood pressure; TC: total cholesterol; TG: triglyceride; LDL-C: low-density lipoprotein cholesterol; hs-cTnI: high-sensitive-cardiac troponin I; ALT: alanine aminotransferase; AST: aspartate aminotransferase. *p < 0.05 vs control.

### 3.2 Identification of the plasma-derived exosomes

**FIGURE 1:**
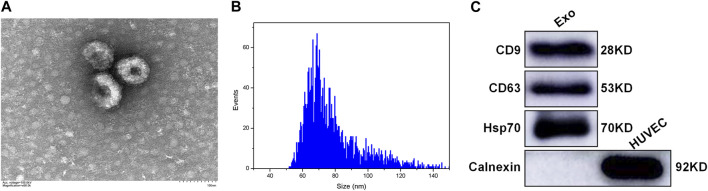
Characterization of the plasma-derived exosomes **(A)** TEM showing the morphological structure of the extracted exosomes **(B)** NTA showing the size distribution of the extracted exosomes **(C)** Western blotting showing the expression of exosomal markers in the extracted exosomes, including CD9, CD63, Hsp70, and Calnexin. Exo: exosome; HUVEC: human umbilical vein endothelial cell; TEM: transmission electron microscope; NTA: nanoparticle tracking analysis.

The plasma-derived exosomes were identified and validated in terms of morphological and biomarker features. Transmission electron microscope images showed that the isolated exosomes were characterized by a tea tray-like bilayer membrane structure (Figure 1A). The data of nanoparticle tracking analysis revealed that the proportion of vesicles with a diameter ranging from 30 to 150 nm was 99.60%, which is consistent with the typical size arrange of exosomes (Figure 1B). The results of western blotting indicated that the extracted exosomes positively expressed CD9, CD63 and Hsp70, but negatively expressed Calnexin (Figure 1C). The above results demonstrated that the majority of the extracted vesicles were exosomes and were pure enough for subsequent experiments.

### 3.3 Plasma exosomal DEmiRNAs between SCAD group and control group by small RNA-Seq

**FIGURE 2:**
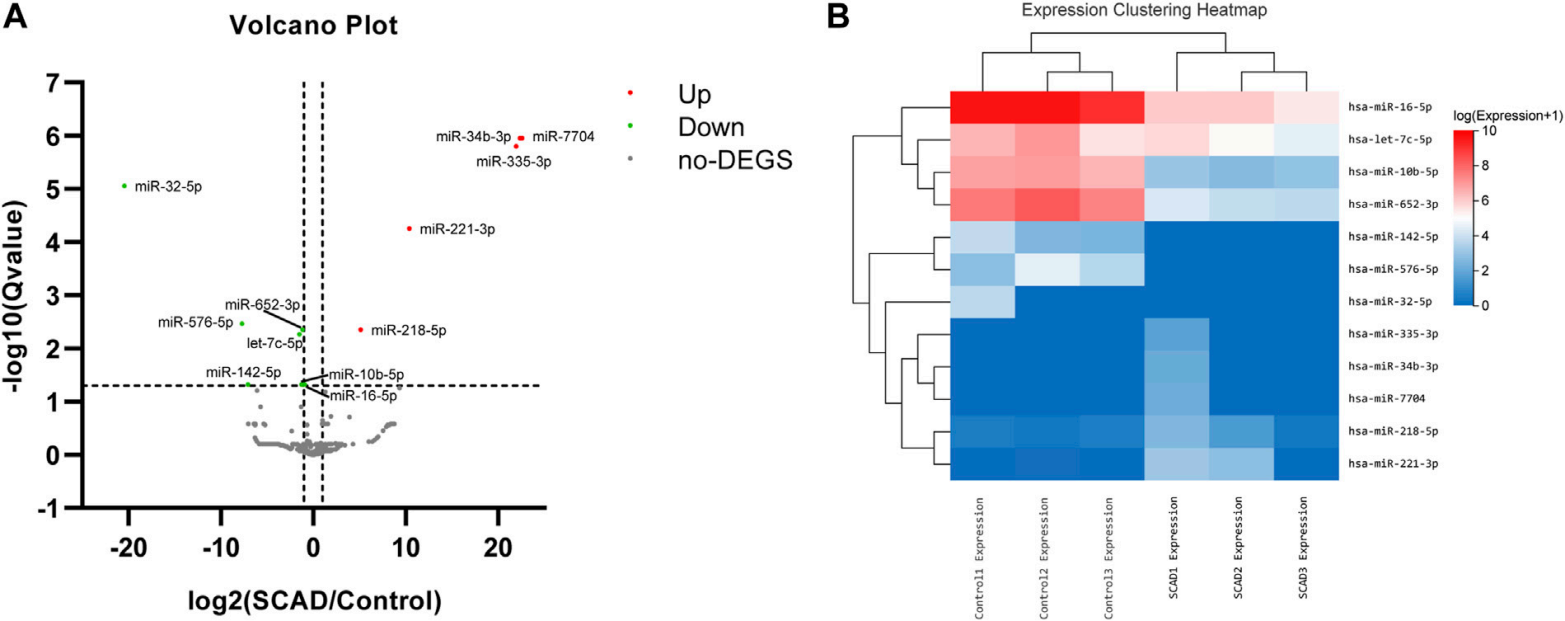
Exosomal DEmiRNAs obtained from small RNA sequencing, a total of 12 DEmiRNAs were found between SCAD patients and healthy controls **(A)** Volcano plot showing the statistically significant DEmiRNAs in the plasma exosomes of SCAD patients (n = 3) compared with control subjects (n = 3). The red and green points represent exosomal DEmiRNAs upregulated and downregulated with statistical significance, respectively **(B)** Heat map and hierarchical cluster analysis showing the relative expression of the exosomal DEmiRNAs. SCAD: stable coronary artery disease; no-DEGS: not differentially expressed genes; DEmiRNAs: differentially expressed miRNAs.

To identify alterations of miRNA expression in plasma exosomes, a small RNA-Seq was first carried out using plasma exosomal miRNA samples isolated from 3 SCAD patients and 3 age-and sex-matched healthy controls. As shown in the expression clustering heatmap (Supplementary Figure S1), a total of 314 miRNAs were identified among the raw data. As illustrated in the volcano plot (Figure 2A), among the 314 identified miRNAs, 12 were found to be differentially expressed, with seven downregulated and five upregulated. The heat map and hierarchical cluster analysis showed the relative expression of the DEmiRNAs between SCAD patients and healthy controls (Figure 2B). The log2 (fold changes: SCAD/Control), *p* and *q* value of each exosomal DEmiRNA are shown in Supplementary Table S2.

### 3.4 Verification of the exosomal DEmiRNAs by qRT-PCR

**FIGURE 3:**
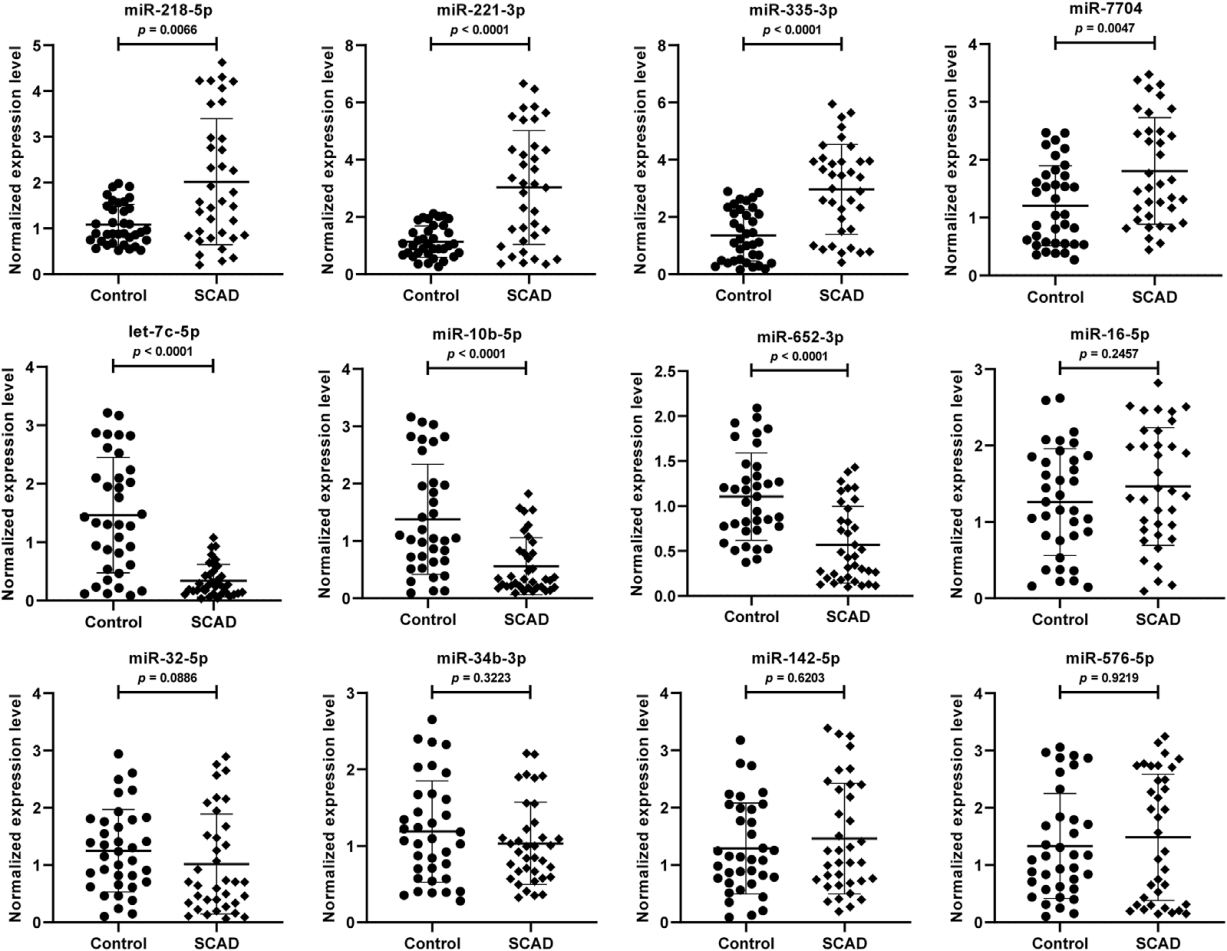
Verification of the 12 DEmiRNAs by qRT-PCR in plasma exosomal miRNA samples of 36 SCAD patients and 36 healthy controls. The relative expression levels of the DEmiRNAs were normalized to U6 and expressed as 2^−ΔΔCT^. SCAD: stable coronary artery disease; DEmiRNAs: differentially expressed miRNAs.

To further validate the 12 DEmiRNAs obtained from the small RNA-Seq, we performed qRT-PCR in the plasma exosomal miRNA samples of SCAD patients (n = 36) and healthy controls (n = 36). As shown in Figure 3, seven of the 12 DEmiRNAs were found to be differentially expressed. Compared with healthy controls, the levels of miR-218–5p, miR-221–3p, miR-335–3p, and miR-7704 were significantly upregulated and let-7c-5p, miR-10b-5p, and miR-652–3p were significantly downregulated in plasma exosomes of SCAD patients (*p* < 0.01). Nevertheless, no significant difference for miR-16–5p, miR-32–5p, miR-34b-3p, miR-142–5p and miR-576–5p between SCAD patients and healthy controls were observed (*p* > 0.05).

### 3.5 ROC curve analysis of the exosomal DEmiRNAs

**FIGURE 4:**
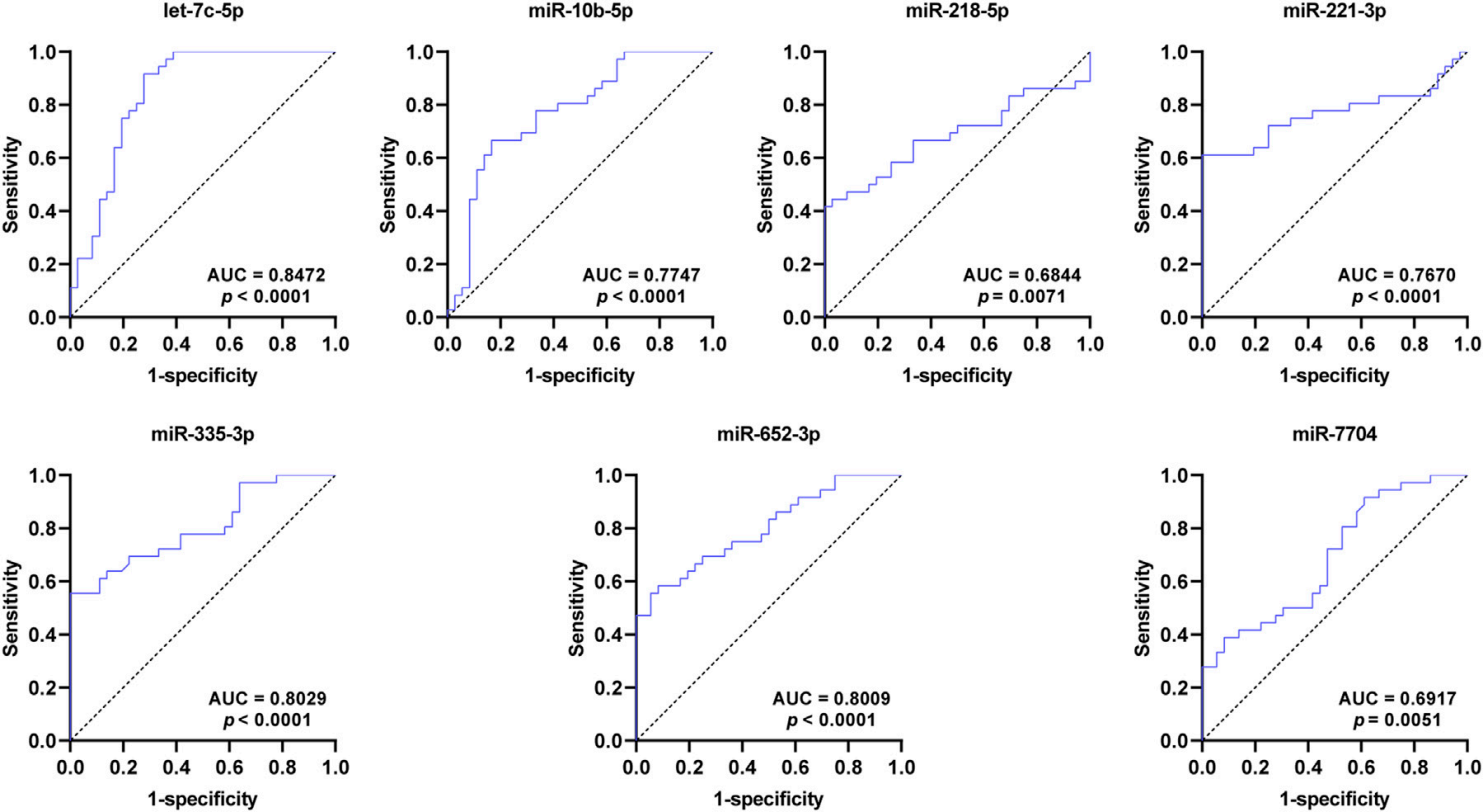
ROC curves of the DEmiRNAs verified by qRT-PCR. The AUC values of exosomal let-7c-5p, miR-335–3p, and miR-652–3p are greater than 0.8. ROC: receiver operating characteristic; AUC: area under the curve; DEmiRNAs: differentially expressed miRNAs.

To assess the diagnostic value for SCAD, ROC curves were further constructed for the seven DEmiRNAs verified by qRT-PCR. As shown in Figure 4 and Table 2, the AUC values of exosomal let-7c-5p, miR-335–3p, miR-652–3p, miR-10b-5p, miR-218–5p, miR-221–3p, and miR-7704 were 0.8472, 0.8029, 0.8009, 0.7747, 0.6844, 0.7670, and 0.6917, respectively, indicating that exosomal let-7c-5p, miR-335–3p, and miR-652–3p whose AUC values are greater than 0.8 may serve as the best biomarkers for SCAD diagnosis.

**TABLE 2:** AUC analysis of the DEmiRNAs verified by qRT-PCR.

miRNA	AUC	Sensitivity (%)	Specificity (%)	95% CI	*p*-value
hsa-miR-652–3p	0.8009	58	92	(0.7005, 0.9013)	<0.0001
hsa-let-7c-5p	0.8472	91	72	(0.7534, 0.9411)	<0.0001
hsa-miR-218–5p	0.6844	44	97	(0.5557, 0.8131)	= 0.0071
hsa-miR-221–3p	0.7670	61	97	(0.6482, 0.8858)	<0.0001
hsa-miR-7704	0.6917	39	92	(0.5707, 0.8128)	= 0.0051
hsa-miR-10b-5p	0.7747	67	83	(0.6650, 0.8843)	<0.0001
hsa-miR-335–3p	0.8029	64	86	(0.7014, 0.9043)	<0.0001

AUC: area under the curve; DEmiRNAs: differentially expressed miRNAs; SCAD: stable coronary artery disease; CI: confidence interval.

### 3.6 Correlation analyses between plasma exosomal let-7c-5p, miR-335–3p, miR-652–3p and genders in patients with SCAD

To further investigate the potential relationships between plasma exosomal let-7c-5p, miR-335–3p, miR-652–3p and genders in patients with SCAD, we performed correlation analyses between these three DEmiRNAs and genders. As shown in Table 3, plasma exosomal let-7c-5p levels were positively correlated with that of miR-652–3p in patients with SCAD (r = 0.434, *p* = 0.008). However, the expression levels of miR-335–3p were not related to that of let-7c-5p (r = −0.056, *p* = 0.747) and miR-652–3p (r = −0.113, *p* = 0.512) in plasma exosomes of patients with SCAD. These results indicated that let-7c-5p and miR-652–3p worked together and miR-335–3p worked independently to act as biomarkers for SCAD diagnosis. Furthermore, the levels of these three DEmiRNAs did not significantly differ between different genders, indicating that these three DEmiRNAs had no prone to genders.

**TABLE 3:** Correlation analyses between plasma exosomal let-7c-5p, miR-335–3p, miR-652–3p and genders in patients with SCAD.

Variable	Let-7c-5p	miR-335–3p	miR-652–3p
let-7c-5p	−	r = −0.056, *p* = 0.747	r = 0.434, *p* = 0.008
miR-335–3p	r = −0.056, *p* = 0.747	−	r = −0.113, *p* = 0.512
miR-652–3p	r = 0.434, *p* = 0.008	r = −0.113, *p* = 0.512	−
Gender	Z = −0.470, *p* = 0.638	Z = −0.973, *p* = 0.330	Z = −0.470, *p* = 0.638

### 3.7 Positive correlation between plasma exosomal miR-335–3p levels and severity of SCAD

**FIGURE 5:**
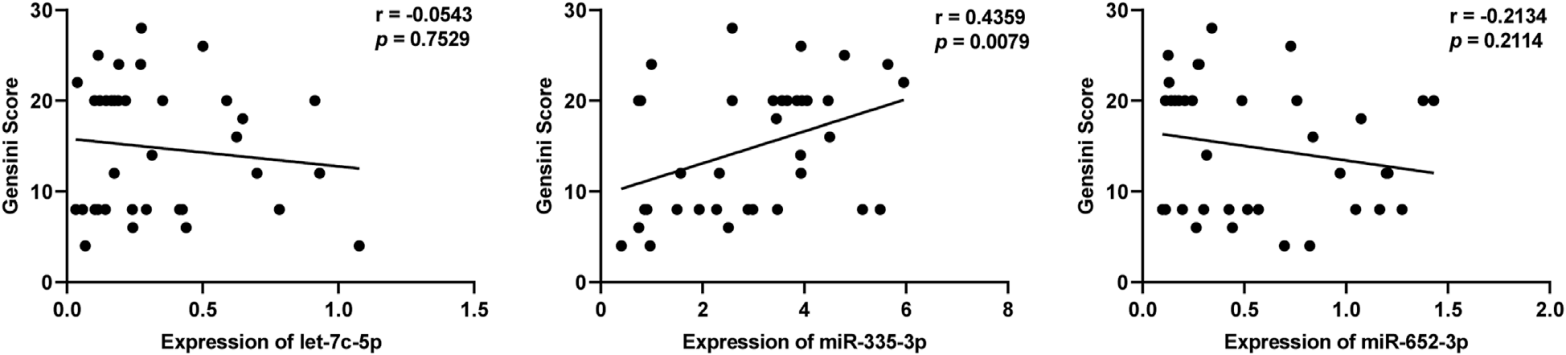
Correlation of plasma exosomal let-7c-5p, miR-335–3p, and miR-652–3 levels with Gensini scores in patients with SCAD.

We further analyzed whether these three DEmiRNAs mentioned above were related to Gensini Scores representing the severity of coronary artery stenosis in patients with SCAD. As shown in Figure 5, the levels of plasma exosomal miR-335–3p (r = 0.4359, *p* = 0.0079), but not let-7c-5p (r = −0.0543, *p* = 0.7529) and miR-652–3p (r = −0.2134, *p* = 0.2114), were positively correlated with Gensini Scores of the SCAD patients, suggesting that miR-335–3p levels coordinated with severity of SCAD.

### 3.8 Bioinformatics analysis of the DEmiRNAs

**FIGURE 6:**
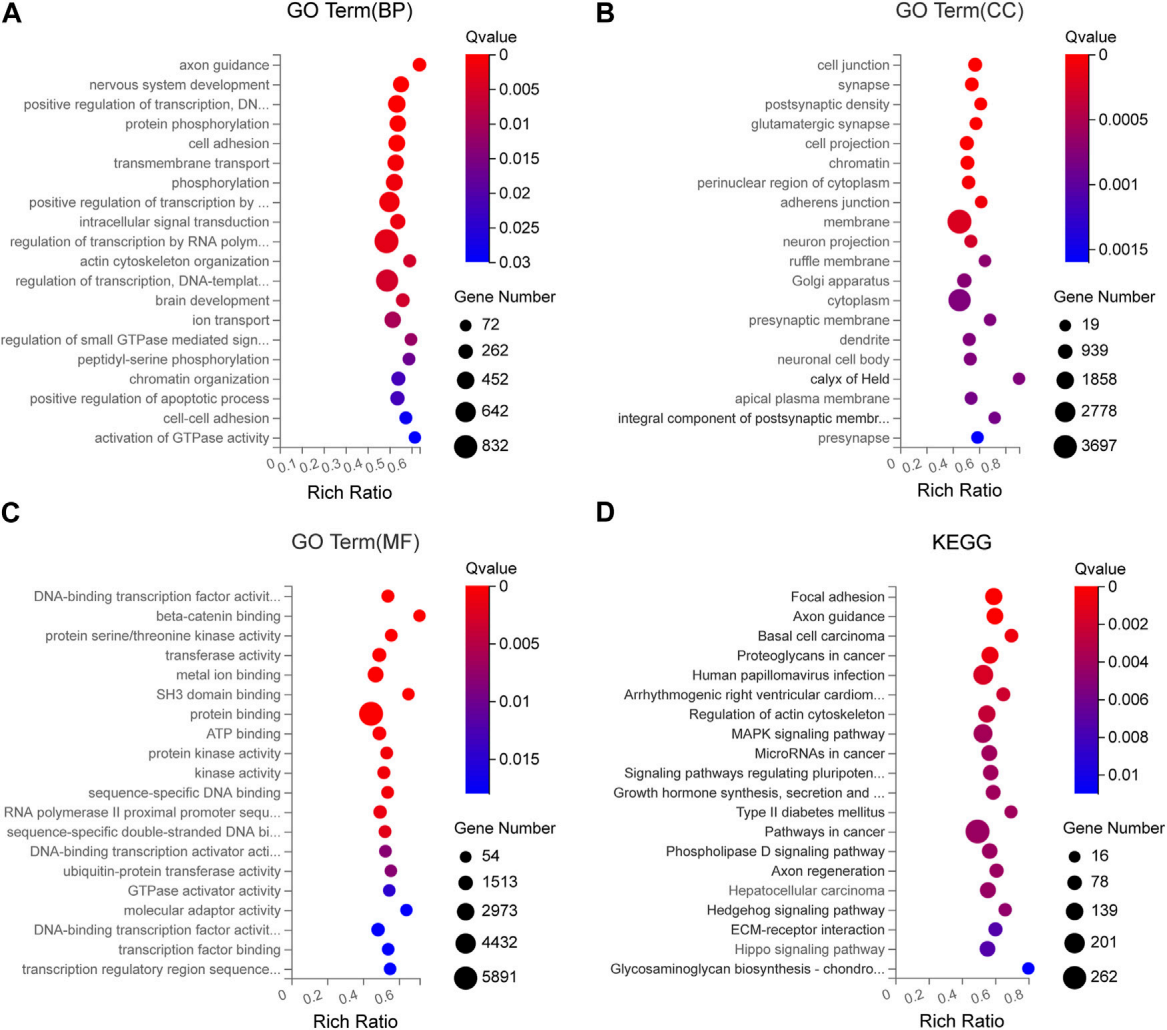
GO and KEGG enrichment analyses for the predicted target genes of DEmiRNAs verified by qRT-PCR **(A)** GO enrichment analysis showing the top 20 most enriched BP related to the target genes **(B)** GO enrichment analysis showing the top 20 most enriched CC related to the target genes **(C)** GO enrichment analysis showing the top 20 most enriched MF related to the target genes **(D)** KEGG enrichment analysis on the predicted target genes. BP: biology process; CC: cellular component; MF: molecular function; GO: Gene Ontology; KEGG: Kyoto Encyclopedia of Genes and Genomes; DEmiRNAs: differentially expressed miRNAs.

To analyze the potential function of the seven DEmiRNAs verified by qRT-PCR in the pathogenesis of SCAD, we identified the predicted target genes of these DEmiRNAs and conducted Gene Ontology (GO) and Kyoto Encyclopedia of Genes and Genomes (KEGG) pathway enrichment analyses for these genes. As shown in Supplementary Table S3, some of the predicted target genes were jointly targeted by at least 2 different DEmiRNAs validated. In the GO enrichment analysis, the predicted target genes generally functioned in protein phosphorylation, cell adhesion, transmembrane transport, *β*-catenin binding, protein serine/threonine kinase activity, transferase activity, metal ion binding, cell junction, synapse, postsynaptic density, and other GO categories (Figures 6A–C). Pathway enrichment analysis showed that the signaling pathways of these target genes include focal adhesion, axon guidance, arrhythmogenic right ventricular cardiomyopathy, regulation of actin cytoskeleton, MAPK signaling pathway, type II diabetes mellitus, ECM-receptor interaction, Hippo signaling pathway and so forth (Figure 6D), most of which were reported to participate in the pathogenesis of CAD, indicating that these DEmiRNAs might play crucial roles in the pathogenetic processes of SCAD.

## 4 Discussion

The most vital characteristics of an ideal biomarker for disease diagnosis are stability, sensitivity, specificity, and noninvasiveness (Mori et al., 2019). For several advantages, there are growing researches exploring the possibility of circulating exosomal miRNAs as diagnostic biomarkers for cardiovascular disease. Firstly, previous researches demonstrated that most of the circulating miRNAs were encapsulated in exosomes and could be detected using sensitive and inexpensive detection methods, even after several years of storage (Mori et al., 2019). Secondly, miRNAs are stable in exosomes because of the protection by the lipid bilayer membrane of exosomes (Henning, 2021). Thirdly, miRNAs in exosomes participate in both normal and pathological physiological processes (He et al., 2021), revealing that the abnormal expression of miRNAs in exosomes may represent the pathophysiology of various diseases to a certain extent. Finally, recent evidence demonstrated that compared with circulating miRNAs, miRNAs in circulating exosomes exhibited better sensitivity in disease diagnosis (Jansen et al., 2014; Beg et al., 2017). Therefore, there is enormous potential for miRNAs in circulating exosomes to be used as novel biomarkers for disease diagnosis.

Whether miRNAs in circulating exosomes are differentially expressed in SCAD patients was rarely studied, therefore, further studies are warranted to investigate the possibility of circulating exosomal miRNAs as diagnostic biomarkers for SCAD. In this study, we first investigated the DEmiRNAs in plasma exosomes between SCAD patients and healthy controls, and further validated these DEmiRNAs by qRT-PCR. Our results indicated that plasma exosomal let-7c-5p, miR-335–3p, miR-652–3p, miR-10b-5p, miR-218–5p, miR-221–3p, and miR-7704 were significantly differentially expressed in SCAD patients compared to healthy controls.

In addition to CAG, several methods including computed tomography angiography (CTA), treadmill exercise test (TET), Holter monitoring, and routine ECG are currently used to diagnose SCAD. The sensitivities of these above methods are 0.92, 0.79, 0.65, and 0.29, respectively, and the specificities are 0.75, 0.80, 0.90, and 0.67, respectively (Sun et al., 2013; Ghadrdoost et al., 2015). Interestingly, we found that the sensitivity and specificity (shown in Table 2) of all the DEmiRNAs verified by qRT-PCR were superior to that of ECG, miR-10b-5p, miR-221–3p, miR-652–3p and miR-335–3p were approximately equal to Holter monitoring and TET, let-7c-5p was superior to Holter monitoring and TET, and approximately equal to CTA. Consequently, among the seven DEmiRNAs, let-7c-5p, miR-335–3p, and miR-652–3p whose AUC values are greater than 0.8 are suggested to be the best promising biomarkers for SCAD diagnosis. More importantly, considering the economy and convenience of detection method, the exosomal DEmiRNAs mentioned above might simplify and ameliorate the diagnosis of SCAD. Additionally, our results further showed that plasma exosomal miR-335–3p levels were positively correlated with Gensini scores in SCAD patients, unraveling the potential of plasma-derived exosomal miR-335–3p in the evaluation of severity of coronary artery stenosis. Thus, detecting plasma exosomal miR-335–3p may contribute to a non-invasive diagnosis modality to assess the degree of coronary artery stenosis.

Recent researches have confirmed that miRNAs in circulating exosomes play important roles in the pathological process of CAD (Li et al., 2020a; Geng et al., 2020; Ye et al., 2021; Zhao et al., 2021; Duan et al., 2022). According to previous reports, four of the seven DEmiRNAs identified in our study have been found to participate in the pathogenesis of atherosclerosis, which is the most common pathological substrate of CAD. Recent research demonstrated that miR-221–3p could induce endothelial cell dysfunction in the pathological process of atherosclerosis by inhibiting PGC-1α (Xue et al., 2015). Besides, miR-221–3p could suppress apoptosis and oxidative stress by targeting metalloprotease-22 and disintegrin (Zhuang et al., 2019). It was found that exosomal miR-7704 may be involved in the process of replicative aging, which is highly associated with cardiovascular morbidity and mortality (Nguyen DDN, 2021). In addition, miR-10b-5p could regulate the formation of coronary artery disease by mediating ABO locus (Majid et al., 2019). With respect to miR-652–3p, one report demonstrated that it could promote atherosclerosis by inhibiting endothelial repair gene Cyclin D2 (Huang et al., 2019). Another report suggested that miR-652–3p could promote atherosclerosis by inhibiting TP53 (Liu et al., 2022). However, the relationship between miR-218–5p, let-7c-5p, miR-335–3p and atherosclerosis or CAD remains unclear.

To further predict the potential roles of the exosomal DEmiRNAs in the pathogenesis of SCAD, the target genes of these DEmiRNAs validated were predicted and GO enrichment and KEGG pathway enrichment analyses were performed for functional annotation. As shown in Figure 6, among the top 20 most enriched GO classes and significant pathways, cell junction (Shih et al., 2023), protein phosphorylation (Wei et al., 2021), cell adhesion (Gencer et al., 2022), transmembrane transport (Weinberg, 2022), *β*-catenin binding (Gao et al., 2019), MAPK signaling pathway (Simion et al., 2020), and Hippo signaling pathway (Xu et al., 2020) were demonstrated to participate in the pathogenetic process of atherosclerosis. Additionally, the functions of the predicted genes targeted jointly by different DEmiRNAs validated were mainly enriched in cell junction, protein phosphorylation and cell adhesion. Therefore, the exosomal DEmiRNAs discovered in this study might be involved in the pathogenesis of SCAD by affecting the biological functions and pathways mentioned above. In our future study, we will focus on investigating the molecular function of these DEmiRNAs in the pathogenesis of SCAD. Overall, these findings may provide new ideas for the future research of the pathogenesis of SCAD and possible targets for the treatment of SCAD.

Despite these strengths, our study has some limitations that should be noted. For instance, only seven of the 12 DEmiRNAs obtained from small RNA-Seq were verified to be differentially expressed with qRT-PCR, indicating that the small sample size of small RNA-Seq limits the robustness of its results. Consequently, further researches are needed to validate the reliability of our results with a larger set of sample size. In addition, due to methodological limitations, we were unable to determine the source of these plasma exosomes containing DEmiRNAs.

## Data Availability

The datasets presented in this study can be found in online repositories. The names of the repository/repositories and accession number(s) can be found below: https://www.ncbi.nlm.nih.gov/bioproject/PRJNA937422
