# Supplementary material for: Plasma-derived exosomal let-7c-5p, miR-335–3p, and miR-652–3p as potential diagnostic biomarkers for stable coronary artery disease
Source: Front Physiol. 2023 May 9;14:1161612. doi: 10.3389/fphys.2023.1161612 (PMC10203605; doi:10.3389/fphys.2023.1161612)
Supplement: Supplementary file 2 [file Table1.docx]

Supplementary Material

Plasma-derived exosomal let-7c-5p, miR-335-3p, and miR-652-3p as potential diagnostic biomarkers for stable coronary artery disease

Jian Han^1†^, Xiaogang Cui^3, 4†^, Tianqi Yuan^3, 4^, Zhiming Yang^2^, Yue Liu^3^, Yajuan Ren^2^, Changxin Wu^3, 4^* and Yunfei Bian^2^*

*** Correspondence:** Changxin Wu: cxw20@sxu.edu.cn (C.W.); Yunfei Bian: [yunfeibian@sina.com](mailto:yunfeibian@sina.com) (Y.B.)

# Supplementary Figures


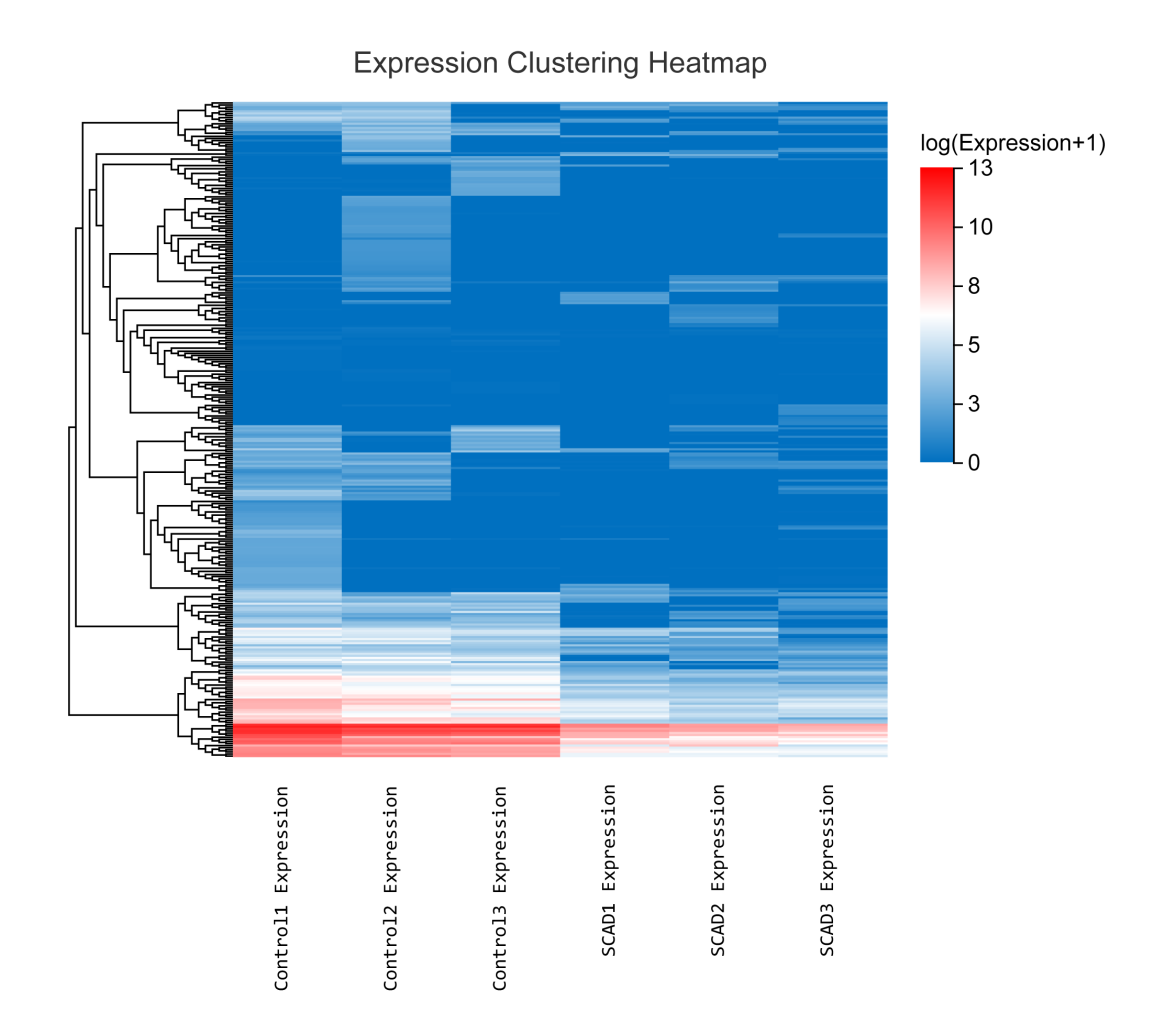


**Supplementary Figure S1.** Expression clustering heatmap of all the exosomal miRNAs identified in small RNA sequencing between SCAD patients (n = 3) and healthy controls (n = 3). SCAD: stable coronary artery disease.

# Supplementary Tables

## Supplementary Table S1. List of the miRNA sequences used for qRT-PCR.

| miRNA | Mature miRNA Sequence |
| --- | --- |
| hsa-let-7c-5p | UGAGGUAGUAGGUUGUAUGGUU |
| hsa-miR-10b-5p | UACCCUGUAGAACCGAAUUUGUG |
| hsa-miR-16-5p | UAGCAGCACGUAAAUAUUGGCG |
| hsa-miR-32-5p | UAUUGCACAUUACUAAGUUGCA |
| hsa-miR-34b-3p | CAAUCACUAACUCCACUGCCAU |
| hsa-miR-142-5p | CAUAAAGUAGAAAGCACUACU |
| hsa-miR-218-5p | UUGUGCUUGAUCUAACCAUGU |
| hsa-miR-221-3p | AGCUACAUUGUCUGCUGGGUUUC |
| hsa-miR-335-3p | UUUUUCAUUAUUGCUCCUGACC |
| hsa-miR-576-5p | AUUCUAAUUUCUCCACGUCUUU |
| hsa-miR-652-3p | AAUGGCGCCACUAGGGUUGUG |
| hsa-miR-7704 | CGGGGUCGGCGGCGACGUG |

## Supplementary Table S2. The [statistical result](javascript:;)s of DEmiRNAs obtained from small RNA sequencing between SCAD patients and healthy controls.

| miRNA | log2 (FC) | *p* value | *q* value |
| --- | --- | --- | --- |
| hsa-miR-218-5p | 5.1159 | 1.35 × 10^−4^ | 0.45 × 10^−2^ |
| hsa-miR-221-3p | 10.3872 | 1.23 × 10^−6^ | 5.54 × 10^−5^ |
| hsa-miR-335-3p | 21.9528 | 2.03 × 10^−8^ | 1.59 × 10^−6^ |
| hsa-miR-34b-3p | 22.3712 | 1.07 × 10^−8^ | 1.12 × 10^−6^ |
| hsa-miR-7704 | 22.4119 | 1.01 × 10^−8^ | 1.12 × 10^−6^ |
| hsa-miR-16-5p | -0.9351 | 0.20 × 10^−2^ | 4.78 × 10^−2^ |
| hsa-miR-652-3p | -1.1368 | 1.42 × 10^−4^ | 0.45 × 10^−2^ |
| hsa-miR-10b-5p | -1.2808 | 0.21 × 10^−2^ | 4.78 × 10^−2^ |
| hsa-let-7c-5p | -1.5025 | 1.89 × 10^−4^ | 0.54 × 10^−2^ |
| hsa-miR-142-5p | -7.0744 | 0.20 × 10^−2^ | 4.78 × 10^−2^ |
| hsa-miR-576-5p | -7.7167 | 8.80 × 10^−5^ | 0.35 × 10^−2^ |
| hsa-miR-32-5p | -20.4444 | 1.68 × 10^−7^ | 8.80 × 10^−6^ |

DEmiRNAs: [differentially expressed](javascript:;) miRNAs; SCAD: stable coronary artery disease; FC: fold change (SCAD/Control).
